# Rosmarinic Acid Inhibits Mitochondrial Damage by Alleviating Unfolded Protein Response

**DOI:** 10.3389/fphar.2022.859978

**Published:** 2022-05-16

**Authors:** Guoen Cai, Fabin Lin, Dihang Wu, Chenxin Lin, Huiyun Chen, Yicong Wei, Huidan Weng, Zhiting Chen, Minxia Wu, En Huang, Zucheng Ye, Qinyong Ye

**Affiliations:** ^1^ Department of Neurology, Fujian Medical University Union Hospital, Fujian Key Laboratory of Molecular Neurology, Institute of Clinical Neurology, Institute of Neuroscience, Fujian Medical University, Fuzhou, China; ^2^ Department of Clinical Medicine, Fujian Medical University, Fuzhou, China; ^3^ Fujian Province Key Laboratory of Environment and Health, School of Public Health, Fujian Medical University, Fuzhou, China; ^4^ College of Pharmacy, Fujian University of Traditional Chinese Medicine, Fuzhou, China; ^5^ Public Technology Service Center, Fujian Medical University, Fuzhou, China; ^6^ Fujian Key Laboratory of Brain Aging and Neurodegenerative Diseases, The School of Basic Medical Sciences, Fujian Medical University, Fuzhou, China

**Keywords:** rosmarinic acid, mtUPR, mitochondrial damage, substantia nigra, immunlogy

## Abstract

Mitochondria are essential organelles that perform important roles in cell biologies such as ATP synthesis, metabolic regulation, immunomodulatory, and apoptosis. Parkinson’s disease (PD) is connected with mitochondrial neuronal damage related to mitochondrial unfolded protein response (mtUPR). Rosmarinic acid (RA) is a naturally occurring hydroxylated polyphenolic chemical found in the Boraginaceae and the Labiatae subfamily Nepetoideae. This study looked into RA’s protective effect against mitochondrial loss in the substantia nigra (SN) caused by 1-methyl-4-phenyl-1,2,3,6-tetrahydropyridine (MPTP), the underlying mechanism associated with the mtUPR. Pretreatment with RA reduced motor impairments and dopaminergic neuronal degeneration in the SN of a mouse model injected with MPTP. Pretreatment of SH-SY5Y cells from cell viability loss, morphological damage, and oxidative stress. Furthermore, RA pre-injection suppressed MPTP-induced mtUPR, lowered the expression of HSPA9, HSPE1, CLPP, LONP1, and SIRT 4, and protected the MPTP-mice and SH-SY5Y cells from mitochondrial failure. These findings imply that RA can prevent Parkinson’s disease by preventing mitochondrial damage in dopaminergic neurons in Parkinson’s disease via alleviating mitochondrial unfolded protein response.

## Introduction

The mitochondria are pivotal organelle and play significant roles in cell biology, including ATP production, metabolic homeostasis, and apoptosis. The coordinated state of complex mitochondria proteome is the basis for mitochondria to perform their normal function. However, proteins are vulnerable to misfolding and thus damaged and aggregated. Oxidative stress is generated in mitochondria. The import of proteins into mitochondria involves the unfolding and refolding of approximately 1,500 nuclear-encoded mitochondrial proteins when they pass through the two mitochondrial membranes (Andreux et al., 2013). The misfolding of mitochondrial proteins can cause ATP deficiency, superoxide anion overload, and an increase in the content of proapoptotic molecules, finally leading to cell death. Thus, the damage of neuronal mitochondria is associated with a series of neurodegenerative diseases, such as Parkinson’s disease (PD), Alzheimer’s disease (AD), and hereditary spastic paraplegia (HSP). Cells respond to the accumulation of unfolded, misfolded, or invalid protein by conducting protein quality control (PQC) and upregulating the expression of nuclear-encoded mitochondrial chaperone proteins such as the heat shock proteins HSPE1 and HSPA9 and proteases such as CLPP, LONP1, and YME1L1), which is known as mitochondrial unfolded protein response (mtUPR) (Tatsuta and Langer, 2008). Chaperones repair the misfolded proteins and assist new synthetic proteins in folding correctly, and proteases degrade invalid proteins (Fiorese and Haynes, 2017).   Therefore, mtUPR synchronizes the activity of mitochondrial and nuclear genomes and ensures that the quality of mitochondrial proteome is maintained.

PD is currently the second-most common neurodegenerative disorder with characteristic features including the neuronal loss in specific areas of substantia nigra (SN) and widespread intracellular protein (α-synuclein) accumulation (Poewe et al., 2017). The pathogenesis of PD has not been fully elucidated; therefore, no neuroprotective drug significantly affects PD. Nevertheless, mitochondrial dysfunction is closely related to PD. Thus, drugs aimed to repair mitochondrial function are the current focus of PD treatment.

Rosmarinic is an evergreen shrub indigenous to the Mediterranean and South America and contains various polyphenols. Rosmarinic extract and its polyphenolic constituents exhibit antioxidant, anti-inflammatory, anticancer, and antihyperglycemic properties.

Rosmarinic acid (RA) is a natural hydroxylated polyphenolic compound widely found in Boraginaceae and subfamily Nepetoideae of the Labiatae. RA exhibits diverse biological activities, including antiviral, antibacterial, antioxidant, antimutagenic, and anti-inflammatory (Elufioye and Habtemariam, 2019). The pharmacological mechanism of RA against PD has not been fully elucidated (Wang et al., 2015). RA exhibited a neurorescue effect by decreasing the nigral iron levels and regulating the ratio of Bcl-2/Bax gene expression (Lv et al., 2019). RA increased the content of Th-positive cells in the SN by reducing inflammation and inhibiting oxidative stress. In this study, we focused on RA’s protection of dopamine neurons in the SN by reducing the occurrence of mtUPR.

MPTP (1-methyl-4-phenyl-1,2,3,6-tetrahydropyridine) is a neurotoxin that activates mtUPR and thus can be used to create PD models. Its active composition, MPP^+^, can be selectively absorbed by dopaminergic neurons and concentrated in the mitochondria, thus inhibiting the electron transport chain (ETC), causing oxidative stress and mtUPR, and finally leading to a series of PD symptoms (Sun et al., 2018). Thus, MPTP is currently used to create experimental PD models in mice.

In this study, MPTP was used to elucidate the neurorescue effect of RA on MPTP-lesioned nigral dopamine neurons in a mice model of PD. In addition, we measured Th-positive cells and a series of heat shock proteins and proteases in SN dopaminergic neurons to establish the relationship between various concentrations of RA and mtUPR induced by MPTP, and then the neuroprotective mechanism of RA on PD is elucidated.

## Materials and Methods

### Animals

This study was performed on male C57BL/6 mice aged 14–16 weeks and weighing 25–28 g, the mice were supplied by the Experimental Animal Center of Fujian Medical University, and the study was approved by the IACUC of FJMU (ethics protocol#number was FJMUIACUC 2020-0015). The animals were housed under standard vivarium conditions (12-h dark/light cycle, 22 ± 1°C ambient temperature) with an *ad libitum* access to food and water. All efforts were made to minimize animal suffering and reduce the number of animals used.

Animals were randomly assigned to following groups (n = 10 per group):

1. Control group: No intervention;
2. NS group: Normal saline (NS) intraperitoneal injection (i.p.);
3. MPTP group: MPTP 20 mg/kg i.p.;
4. MPTP + 40 mg/kg RA group: MPTP 20 mg/kg + RA 40 mg/kg i.p.;
5. MPTP + 60 mg/kg RA group: MPTP 20 mg/kg + RA 60 mg/kg i.p.;
6. MPTP + 80 mg/kg RA group: MPTP 20 mg/kg + RA 80 mg/kg i.p.

### Rosmarinic Acid Injection

Rosmarinic acid (Yuanye technology, Shanghai, China, Cat# B20862) was administered once a day by i.p. injection of 40, 60, and 80 mg/kg RA, while NS was administered by i.p. injection in the normal saline group (Wei et al., 2021).

### PD Induction

**FIGURE 1:**
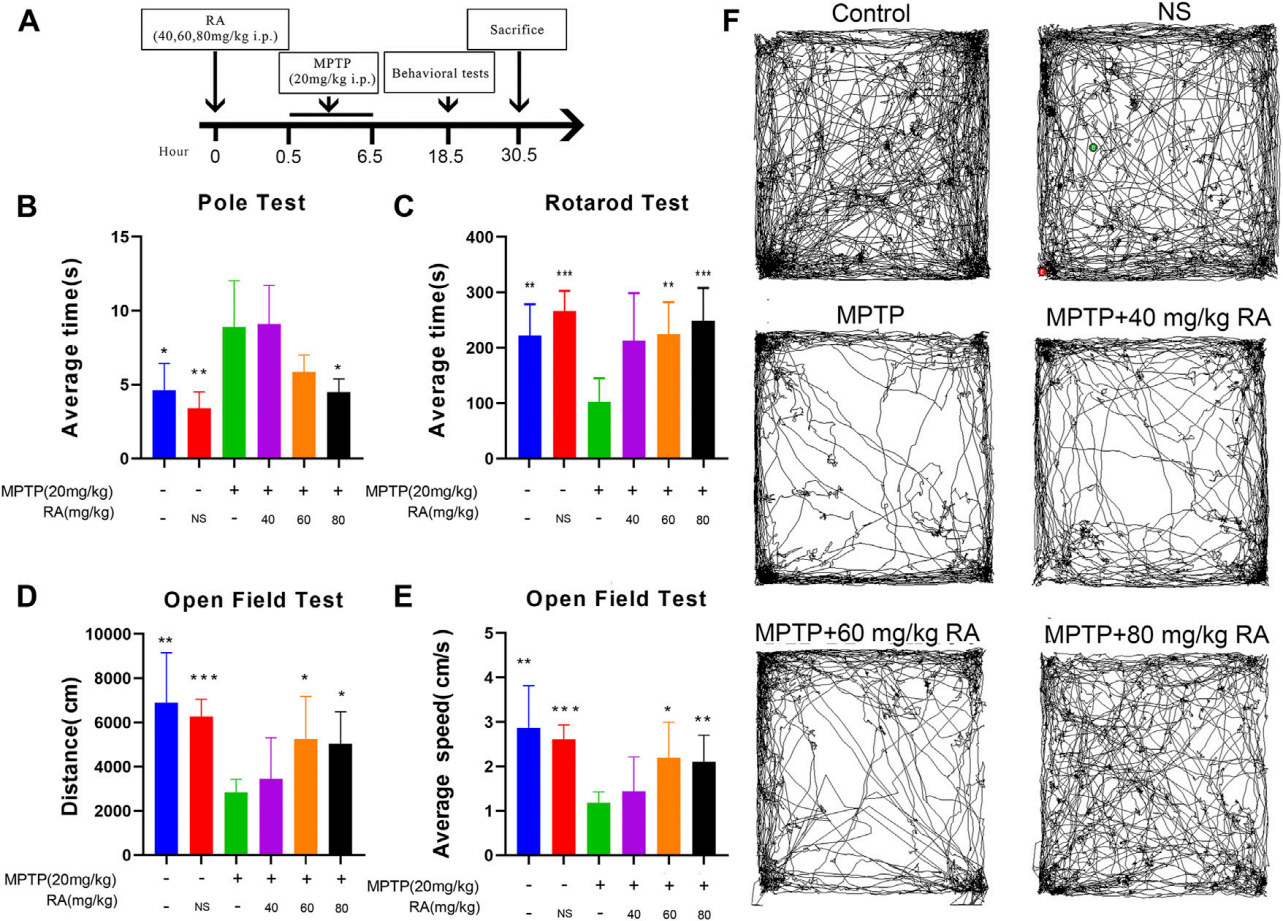
RA pre-administration ameliorated MPTP-induced motor abnormalities in MPTP mice. The experimental schedule for RA intervention in the MPTP-mice model is shown in **(A)**. The pole test **(B)**, rotarod test **(C)**, and open-field test **(D,E)** were used to evaluate the motor function of PD mice. Representative examples of movement path **(F)** were selected. Compared with the control and PBS, the MPTP-injected mice showed a higher climbing time in the pole test, decreased latency in the rotarod test, and decreased movement distance and speed in the open field test. RA shortened the pole test’s climbing time, increased the rotarod test’s latency, and increased movement distance and speed in the open field test. Data are expressed as mean ± SD. One-way analysis of ANOVA with Dunnett’s T3 test was used. N = 10 mice per group, *p < 0.05, **p < 0.01, ***p < 0.001 when compared with the MPTP group.

After 30 min of RA injection, the animals were intraperitoneally injected with MPTP (Sigma-Aldrich, MO, United States, Cat# M0896) four times at a dose of 20 mg/kg at 2-h intervals. The same number of NS was injected into the NS group. The schedule of animal experiments is shown in Figure 1A.

### Cell Preparation

Exponentially growing human neuroblastoma cell line SHSY5Y (Chinese Academy of Sciences Cell Bank, Shanghai, China, Cat#SCSP-5014) were maintained in a 1:1 mixture of Ham’s F12 and Dulbecco Modified Eagle Medium (DMEM) (Sigma-Hyclone, MO, United States, Cat# SH30023.01) supplemented with 10% heat-inactivated FBS (Sigma- Gibco, MO, United States, Cat# 16140089), 100 IU/ml of Penicillin and 100 ug/ml of streptomycin (Sigma- Gibco, MO, United States, Cat#15140148), in a humidified atmosphere of 5% of CO_2_ in the air at 37°C (Thermo Fisher Scientific, MA, United States). Cells were sub-cultured once they reached 80–90% confluence. Rosmarinic acid (20, 40, 60, 80, 100, and 200 μM) and MPP^+^ (400 μM) (Sigma-Aldrich, MO, United States, Cat# M7068) were dissolved in normal saline then added into the culture medium. The cells were seeded into 25 cm^2^ culture flasks for 24 h, then divided into 9 groups of: control, PBS, MPP^+^, MPP^+^ + 20 μM RA, MPP^+^ + 40 μM RA, MPP^+^ + 60 μM RA, MPP^+^ + 80 μM RA, MPP^+^ + 100 μM RA and MPP^+^ + 200 μM RA.

### Motor Performance Test

The mice’s motor performance was assessed using the following experiments: pole test, open-field test (OFT), and rotarod test.

### Pole Test

The pole test is a useful method to measure the bradykinesia and movement balance in a mouse PD model. The pole test was performed on the 1st day after the last MPTP injection. The animals were placed facing upward near the top of a wooden pole (diameter 8 mm, height 50 cm with a rough surface). The time needed for the mice to climb down the pole and place four feet on the floor (defined as locomotor activity time, T-LA) was recorded. The mice were pre-trained three times before MPTP injections. Every mouse was tested three times, and the average of three trials was calculated for statistical analyses (Park et al., 2013).

### Open-Field Test

The ambulatory behavior and exploratory ability were assessed in an open field test conducted on the 2nd day after MPTP injection. The animals were individually placed in the center of an acrylic apparatus (40 cm*40 cm*40 cm). The tracks, hotspots, and average speed, were monitored using Smart 3.0 software (Panlab, Barcelona, Spain). The apparatus was cleaned with 75% ethanol solution and dried between trials to avoid the presence of olfactory cues (Li et al., 2016).

### Rotarod Test

Sensorimotor coordination was assessed with a rotarod test [YLS-4C Rota Rod with automatic timers and falling sensors (Yiyan Scientific, Shangdong, China)]. Animals were evaluated on the rotarod day after MPTP injection and pretrained three times before PD induction. All mice were pretrained for three consecutive days with two trials per day (5 rpm, 1 min → 10 rpm, 2 min → 15 rpm, 3 min) with 15-min intervals between each trial. In the test session, the test rotation speed was increased to 40 rpm, and the length of time taken until the mouse fell from the rod was recorded. Every mouse was tested three times, and the average of three trials was calculated for statistical analyses. The rotarod test was conducted by examiners blinded to the treatment.

### Brain Tissue Preparation

On the third day after MPTP injection, the animals were sacrificed by anesthetic overdose, and the brains were immediately removed with different methods for the following experiments.

### Electron Microscopy

The mice were sacrificed, as mentioned above. The brains were removed, and the SN was dissected and processed with 3% glutaraldehyde (Tianjin Hengxing Chemical Reagent Co., Ltd., Tianjin, China), 1.5% paraformaldehyde (Tianjin Hengxing Chemical Reagent Co., Ltd., Tianjin, China), and 0.1 M PBS (Sigma- Gibco, MO, United States, Cat# 70011069) at 4°C per 24 h each. This procedure was followed by post-fixations in 1% osmium tetroxide and 1.5% potassium ferrocyanide at 4°C for 1.5 h. After washing with PBS, the samples were dehydrated in a graded series of ethanol (75%, 95%, 100% v/v) and embedded in an Epon-Araldite solution (Ted Pella, Redding, CA, United States) at 60°C for 72 h. Then, the sections were cut at 100 nm thickness using an ultramicrotome (Leica, Wetzlar, Germany) and imaged under an EM208 transmission electron microscope (Philips, Eindhoven, the Netherlands).

### Immunohistochemistry

The mice were sacrificed and intracardially perfused with 30 ml of 0.1 M PBS and then fixed with 4% PFA. The brains were removed and post-fixed in 4% PFA at 4°C overnight and then immersed in a solution containing 30% sucrose (BioSharp, Hefei, China, Cat# BS127A) 0.1 M PBS for cryoprotection. The tissues were cryo-sectioned at 15 μm thickness using a cryo-microtome (Leica, Wetzlar, Germany) and then placed on coated slides.

### TH Staining

The slides were pretreated with 3% hydrogen peroxide for 15 min to remove endogenous peroxidase activity. The slides were incubated overnight with the rabbit anti TH antibody (1:1,000 dilutions) (Abcam, Cambridge, UK, Cat# ab112) in 5% NGS (Beijing Zhongshan Jinqiao Biotechnology Co., Ltd., Beijing, China, Cat# ZLI-9021) +PBST, rinsed with 0.1 M PBS three times, 5 min each, and then incubated with biotinylated goat anti-rabbit IgG (Beyotime Biotechnology, Shanghai, China, Cat#A0277) at room temperature for 2 h. The second antibody was removed, and the slides were rinsed with 0.1 M PBS and incubated with the avidin-biotin-peroxidase complex reagent at room temperature for 1 h. The samples were colored in 3,3-diaminobenzidine (DAB) solution (Thermo Fisher Scientific, MA, United States, Cat# 34002) for 3–5 min using distilled water to terminate the response. The number of DA (TH positive) neurons was only counted from the sections in the region containing the medial terminal nucleus (MTN) because this region has been previously shown to express the highest level of virus-mediated gene expression after intrastriatal infection. We also used the MTN as a landmark to evaluate consistent levels of SNc (Huang et al., 2017). One section per animal and three animals per group were analyzed. We also did normalization to divide the number of DA neurons by the area (number of DA neurons/area). All the stained sections were viewed and photographed using a light scope to measure the density of TH-positive cells in the SN, and the images were scanned using ImageJ software (ImageJ, NIH, United States) to complete the quantitative analysis of DA neurons.

### Measurement of Mitochondrial Membrane Potential

Following the manufacturer’s instruction, the MMP change was determined using a JC-1 Assay Kit (Beyotime Biotechnology, Shanghai, China, Cat# C2003S). In brief, primary cortical neurons (5 × 10^5^ cells/well) cultured in 6-well plates were harvested. After washing, neurons were resuspended in a 500 μL growth medium and incubated with JC-1 working solution (2 μM final concentration) in the dark at 37°C for 20 min. For positive control, the mitochondrial uncoupler, CCCP (10 μM final concentration), was added simultaneously with JC-1. CCCP is a positive control group in JC-1, which is a powerful uncoupling agent for mitochondrial oxidative phosphorylation, which promotes the permeability of mitochondrial intima to H^+^, resulting in mitochondrial apoptosis. The loss of membrane potential on both sides of the intima induces apoptosis. Samples were then analyzed using a confocal microscope (LSM 750, Zeiss, Gottingen, Germany). Data were processed with the ImageJ software, and the results were represented as the relative ratio of green to red fluorescence intensity.

### Measurement of Intracellular ROS Levels

The intracellular ROS levels were measured using a Reactive Oxygen Species Assay Kit (Beyotime Biotechnology, Shanghai, China, Cat# S0033S) 2′, 7′-dichlorofluorescein-diacetate (DCFH-DA), which is easily oxidized to fluorescent dichlorofluorescein (DCF) by intracellular ROS, is its principal component, and therefore, the ROS levels were quantified. Briefly, the cells were seeded in 96-well plates as described above and exposed to MPP_+_ and various concentrations of RA for different time intervals. The cells were incubated with DCFH-DA for 20 min at 37°C and then observed using a confocal microscope (LSM 750, Zeiss, Gottingen, Germany). Data were processed with the ImageJ software, and the results were represented as the relative ratio of green to red fluorescence intensity.

### Cell Viability

Cell viability was evaluated by an enhanced cell counting kit-8 (CCK-8) assay following the manufacturer’s instruction (Dojindo, Kumamoto, Japan, Cat# CK04-11). In short, SH-SY5Y were seeded in a 96-well plate at a concentration of ×1 104 (200 μL/well) for 7 days. After treatment, add 10 μL of CCK-8 reagent to each well and incubate at 37°C for 2 h. Use a microplate reader (SpectraMax®i3x, Molecular Devices, United States) to measure the sample’s optical density (OD) value at 450 nm.

### Tissue Preparation

Three days after MPTP injection, the animals were sacrificed by anesthetic overdose and intracardially perfused with 30 ml of ice-cold 0.1 M PBS. Each SN was dissected and homogenized on ice in RIPA lysis buffer (Beyotime Biotechnology, Shanghai, China, Cat# P0013) containing phenylmethylsulfonyl fluoride (PMSF) (Beyotime Biotechnology, Shanghai, China, Cat# ST506) for 30 min. Tissue lysates were obtained by centrifugation (Thermo Fisher Scientific, MA, United States) at 12,000 rpm for 5 min at 4°C.

### Western-Blot Analysis

The protein concentration was quantified using bovine serum albumin as the standard by a BCA protein assay (Beyotime Biotechnology, Shanghai, China, Cat# P0012). After tissue lysate of cells was mixed with loading buffer and boiled for 5 min, 30 μg of denatured protein was loaded per lane, resolved by 10% SDS-PAGE (sodium dodecyl sulfate-polyacrylamide gel electrophoresis) (Beyotime Biotechnology, Shanghai, China, Cat# P0012A) for 90 min at 80 V, and then transferred to polyvinylidene difluoride (PVDF) membranes (Millipore, MA, United States, Cat# ISEQ00010). The membranes were blocked with 5% BSA in tris-buffered saline and incubated with primary antibodies (CLPP, HSPA9, HSPE1, LONP1, and SIRT4 for cell specimen; CLPP, HSPA9, HSPE1, LONP1, and SIRT4 for tissue specimen) [HSPE1 (Abcam, Cambridge, UK, Cat# ab181606), HSPA9 (Abcam, Cambridge, UK, Cat# ab129201), CLPP (Abcam, Cambridge, UK, Cat# ab126102), LONP1 (Abcam, Cambridge, UK, Cat# ab103809) and SIRT4 (Proteintech, Wuhan, China, Cat# 66543-1-Ig)] at 4°C overnight, followed by incubation for 90 min at room temperature with secondary antibody conjugated to HRP [HRP-labeled Goat Anti-Rabbit IgG (Beyotime Biotechnology, Shanghai, China, Cat# A0208), HRP-labeled Goat Anti-Mouse IgG (Beyotime Biotechnology, Shanghai, China, Cat# A0216)]. The bound antibodies were then visualized by enhanced chemiluminescence (ECL). Gels were recorded under ultraviolet light with a GelDoc XR system and analyzed with the ImageJ software.

### Statistical Analysis

In this study, the statistical analysis used was one-way ANOVA followed by post hoc comparisons using Tukey’s test or Dunnett’s T3 test for comparisons among multiple groups, Tukey’s test was used when variances were assumed equal, and Dunnett’s T3 test was used when variances were assumed to be unequal across groups, and the data were analyzed with SPSS 24.0 (IBM, Armonk, NY, United States). All data are presented as the mean ± SD. *p* < 0.05 was considered statistically significant.

## Results

### RA Pre-Administration Ameliorated MPTP-Induced Motor Abnormalities in MPTP Mice

The effective behaviors were examined on day 1 post-MPTP (Figure 1B). The animals were then subjected to various motor function tests to detect behavioral dysfunction, including the pole test, rotarod test, and OFT.

In the poletest, the MPTP-injected mice showed a significant prolongation of climbing time compared with the control and PBS (control vs. MPTP, *p <* 0.05; NS *vs.* MPTP, *p <* 0.01; Figure 1B), but this prolongation was significantly improved by RA pre-administration (MPTP *vs.* MPTP+ 80 mg/kg RA, *p <* 0.05; Figure 1B).

When subjected to the rotarod test, the MPTP-injected mice showed a significantly decreased latency-to fall (control *vs.* MPTP, *p <* 0.01; NS *vs.* MPTP, *p <* 0.001; Figure 1C), but the pre-injection with RA effectively alleviated this motor abnormalities (MPTP *vs.* MPTP+ 60 mg/kg RA, *p <* 0.01; MPTP *vs.* MPTP+ 80 mg/kg RA, *p <* 0.001; Figure 1C).

There was a significant decrease in the movement distance of MPTP-injected mice in the OFT (control *vs.* MPTP, *p <* 0.01; NS *vs.* MPTP, *p <* 0.001; Figure 1D), but this deficit was improved with the pre-injection with RA (MPTP *vs.* 60 mg/kg RA, *p <* 0.05; MPTP *vs.* MPTP+ 80 mg/kg RA, *p <* 0.05; Figure 1D). Additionally, a significant decrease was observed in the average movement speed of MPTP-injected mice (control *vs.* MPTP, *p <* 0.01; NS *vs.* MPTP, *p <* 0.001; Figure 1E), but this deficit was improved with the pre-injection with RA (MPTP *vs.* MPTP+ 60 mg/kg RA, *p <* 0.05; MPTP *vs.* MPTP+ 80 mg/kg RA, *p <* 0.01; Figure 1E). Representative examples of movement paths are shown in Figure 1F.

### RA Pre-Administration Prevented the Dopaminergic Neuronal Loss in the SN

**FIGURE 2:**
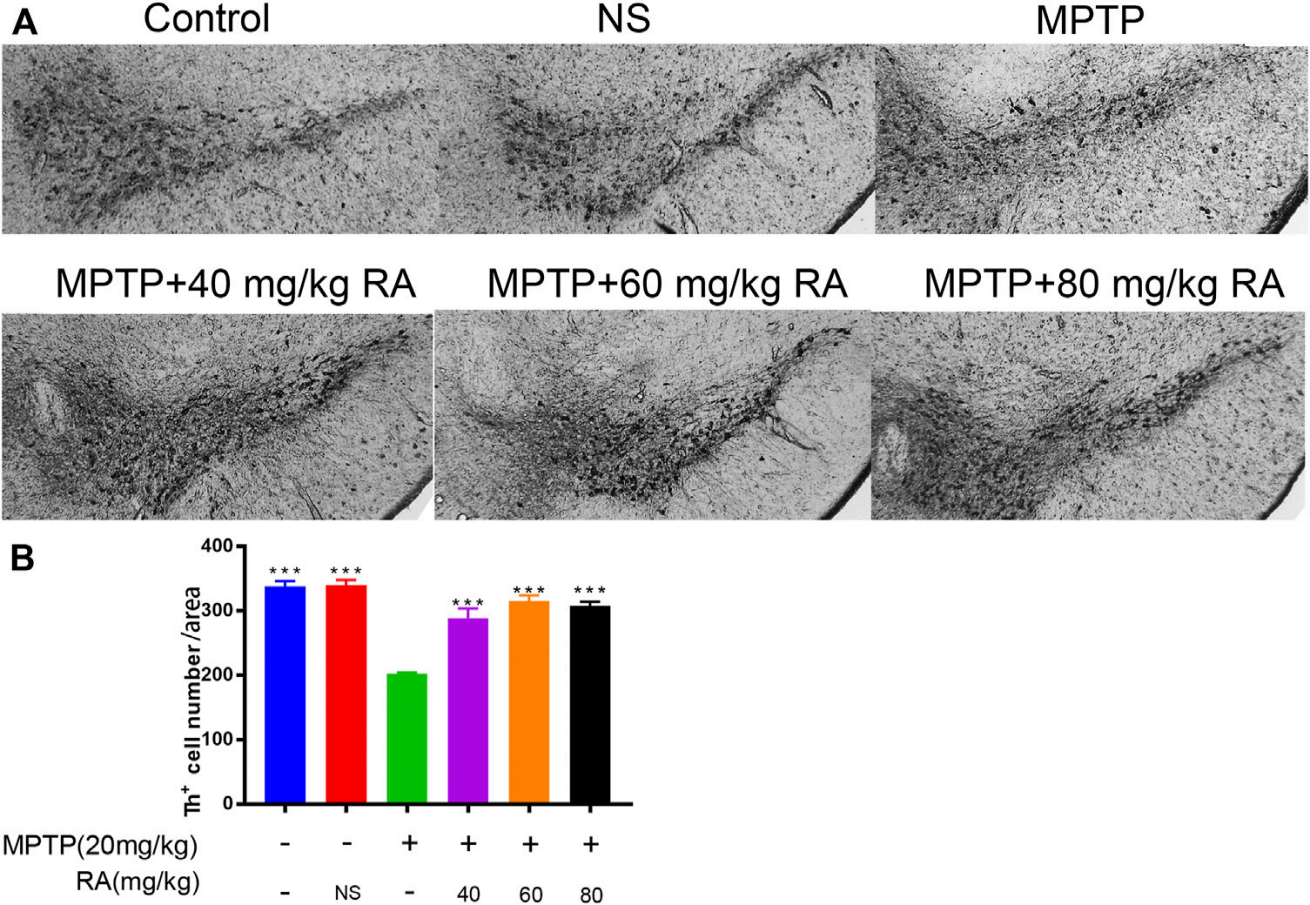
RA prevented the dopaminergic neuronal loss in the SN of PD mice. Compared with the control, MPTP induced 55.7% of dopaminergic neuronal loss in the SN, and this loss was improved by preinjecting with 40 mg/kg RA (induced 7.80%), 60 mg/kg RA (induced 8.30%), and 80 mg/kg RA (induced 6.80%) **(A,B)**. Data are expressed as mean ± SD. One-way analysis of ANOVA with Tukey’s test was used. N = 3 mice per group, *p < 0.05, **p < 0.01, ***p < 0.001 when compared with the MPTP group.

The MPTP-injection showed a significant effect on the dopaminergic neurons in SN (control *vs.* MPTP, *p <* 0.001; NS *vs.* MPTP, *p <* 0.001; Figure 2). Compared with the MPTP injection alone, pre-injection with RA significantly decreased the loss of TH^+^ cells in the SN. Interestingly, no difference was observed between different doses of RA (*p* > 0.05; Figure 2).

### RA Pre-Administration Restored the Mitochondrial Cristae Morphology and Mitochondrial Length Compromised by MPTP Administration

The changes in mitochondrial ultrastructure in MPTP-mice were investigated by transmission electron microscopy 30.5 h after RA administration to evaluate the therapeutic effect of pre-injection of RA on the ultrastructural abnormalities of brain mitochondria after MPTP administration.

**FIGURE 3:**
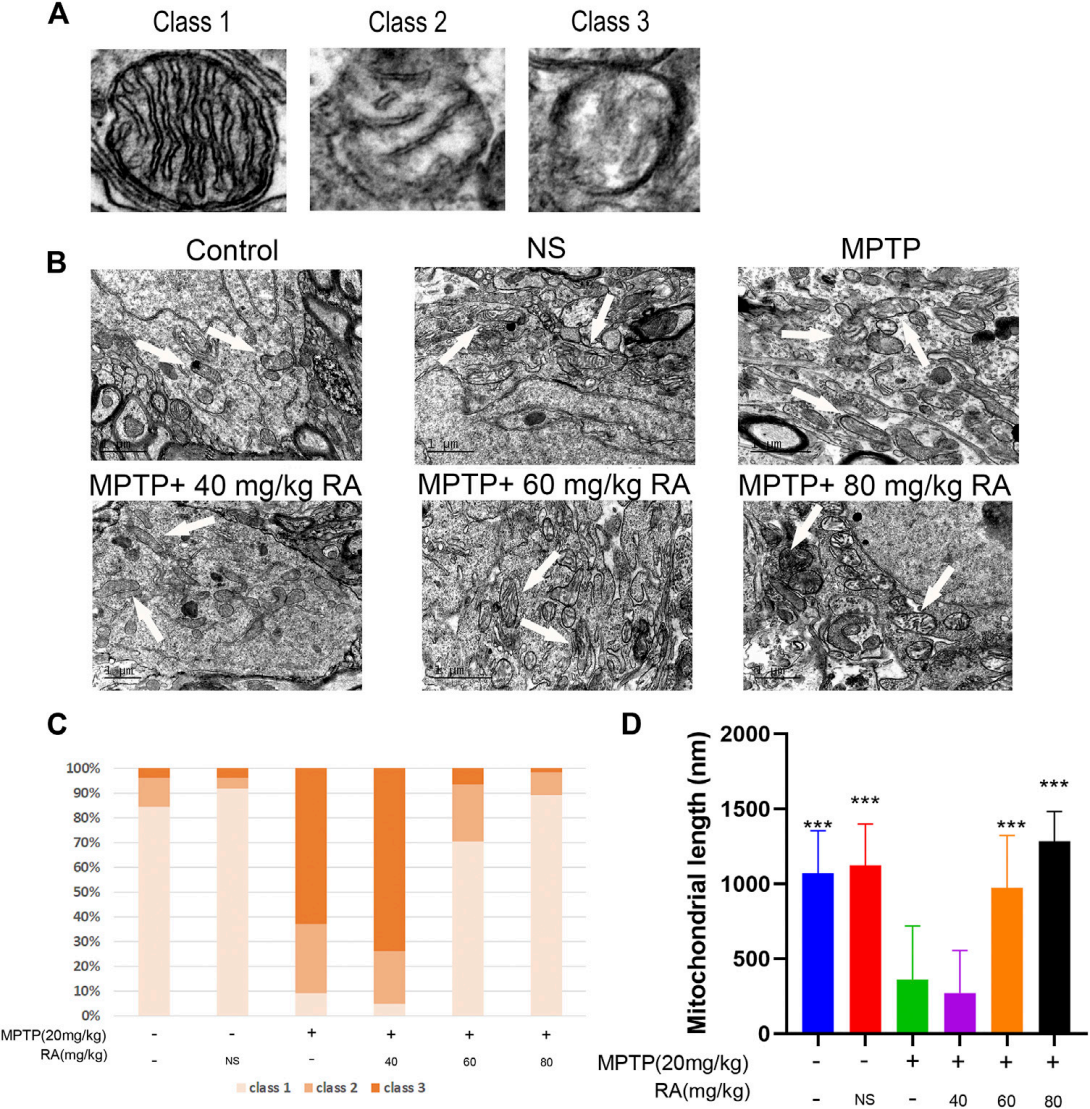
The restored mitochondrial cristae morphology and mitochondrial length after cerebral MPTP injections *in vivo* by RA pre-administration. **(A)** Representative transmission electron microscopy (TEM) images of mitochondrial cristae number and matrix density. **(B)** Representative TEM images in the cortex of different experimental groups. **(C)** 40–70 mitochondria per experiment were scored in three categories: beyond four cristae (Class I), two or three cristae (Class II), no more than one crista (Class III) per mitochondrion. **(D)** Representative quantitative results of mitochondrial length with 40–70 mitochondria per experiment. Data are expressed as mean ± SD. One-way analysis of ANOVA with Tukey’s test was used. N = 3 mice per group, *p < 0.05, **p < 0.01, ***p < 0.001 when compared with the MPTP group.

The mitochondria were categorized into the following types (Del Dotto et al., 2017): more than four cristae (type I), two or three cristae (type II), not more than one crista (type III) (Figures 3A,B). The control group showed 84% type I mitochondria, and the NS group showed 92%. However, the mitochondria suffered extensive damage in the MPTP injury group: about 63% of class III mitochondria, 28% of class II mitochondria, and 9% of class I mitochondria, indicating a dramatic reduction of the mitochondria cristae; meanwhile, the length of mitochondria was significantly shortened. However, RA pre-injection significantly alleviated abnormal mitochondrial ultrastructure, showing 70% of class I mitochondria, 23% of class II mitochondria, and 7% of class III mitochondria in the MPTP+ 60 mg/kg RA group. The mitochondrial length was significantly improved in the MPTP+ 60 mg/kg RA and MPTP+ 80 mg/kg RA group (Figure 3C). These data indicate that RA pre-injection can effectively prevent the ultrastructure of mitochondria from being compromised by MPTP administration.

### RA Alleviated MPP^+^-Induced Oxidative Stress and Mitochondrial Dysfunction in SH-SY5Y Cells

**FIGURE 4:**
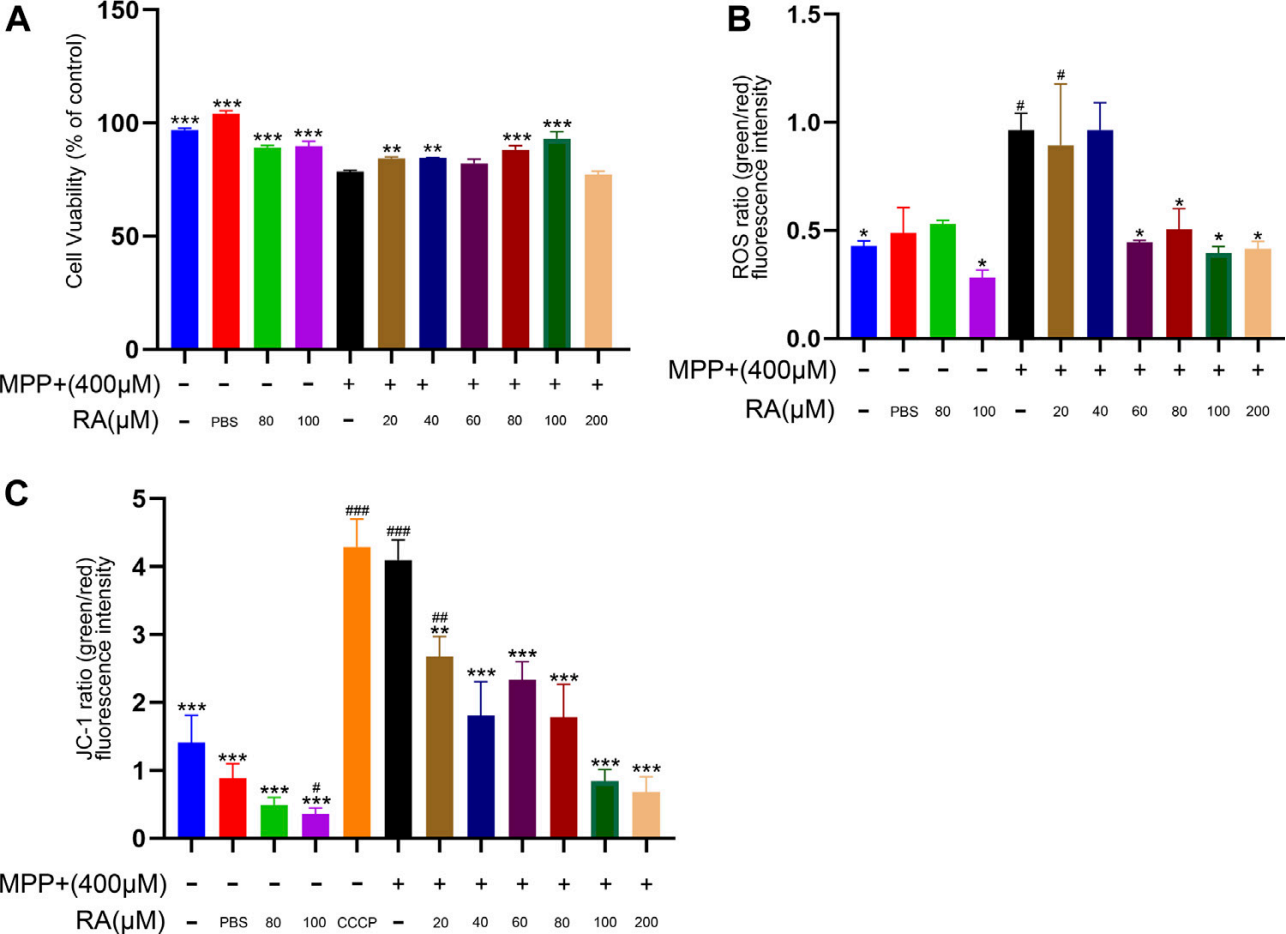
RA protected SHSY5Y cells against MPP+-induced loss of cell viability and oxidative stress. **(A)** SH-SY5Y cells viability was measured by CCK-8 after being incubated with various concentrations of RA. MPP^+^ resulted significantly decrease in cell viability, and pre-injection with RA in a dose-dependent manner significantly reduced the MPP^+^-induced reduction in cell viability. One-way analysis of ANOVA with Tukey’s test was used. Data are expressed as mean ± SD. N = 3 cells per group, *p < 0.05, **p < 0.01, ***p < 0.001 when compared with the MPP^+^ group. **(B)** ROS was utilized to evaluate the oxidative stress of the SH-SY5Y cells after RA incubation. MPP^+^ caused increased the green/red fluorescence intensity in the SH-SY5Y cells, and RA significantly reversed these changes in a dose-dependent manner. One-way analysis of ANOVA with Dunnett’s T3 test was used. Data are expressed as mean ± SD. N = 3 cells per group, *p < 0.05, **p < 0.01, ***p < 0.001 when compared with the MPP^+^ group, ^#^ *p <* 0.05, ^##^ *p <* 0.01, ^###^ *p <* 0.001 when compared with the control group. **(C)** JC-1was utilized to evaluate the mitochondrial membrane potential (MMP) of the SH-SY5Y cells after RA incubation. The green/red fluorescence intensity increased in the MPP^+^ group when stained with JC-1, and this ratio was markedly decreased by preincubation with RA. One-way analysis of ANOVA with Tukey’s test was used. Data are expressed as mean ± SD. N = 3 cells per group, *p < 0.05, **p < 0.01, ***p < 0.001, #*p* < 0.05, ##*p* < 0.01, ###*p* < 0.001 when compared with the control group.

**FIGURE 5:**
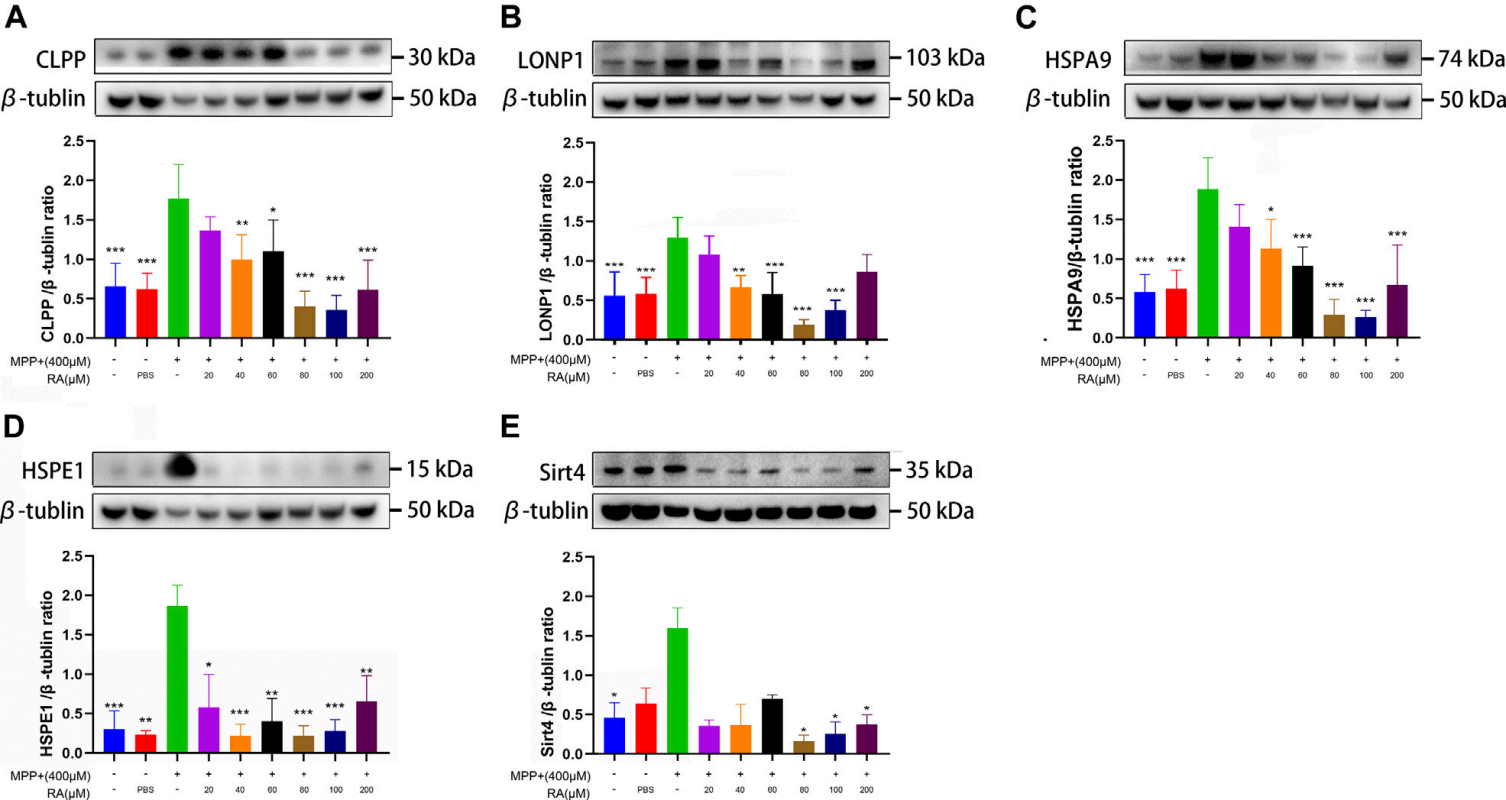
RA alleviated MPP^+^-induced mitochondrial damage via the mtUPR in SH-SY5Y cells. MPP^+^ caused a significant increase in CLPP **(A)**, LONP1 **(B)**, HSPA9 **(C)**, and HSPE1 **(D)** expression, and RA reversed these increases in a dose-dependent manner. RA regulated SIRT4 **(E)** expression in SHSY5Y cells. The expression of SIRT4 was increased by MPP^+^, and RA reversed this increase in a dose-dependent manner. Data are expressed as mean ± SD. One-way analysis of ANOVA with Tukey's test was used in CLPP, LONP1, and HSPA9. One-way analysis of ANOVA with Dunnett's T3 test was used in HSPE1 and Sirt4. N = 3-8 cell samples per group, *p < 0.05, **p < 0.01, ***p < 0.001 when compared with the MPP^+^ group.

To investigate how RA protects against dopaminergic neurodegeneration, SH-SY5Y cells were used to identify the related molecular mechanism. As shown in Figure 4A, MPP^+^ resulted a significantly decrease of cell viability (MPP^+^ *vs.* control, *p <* 0.001; MPP^+^ *vs.* PBS, *p <* 0.001; Figure 4A) and pre-injection with RA significantly reduced the MPP^+^-induced reduction in cell viability in a dose-dependent manner. (MPP^+^ *vs.* MPP^+^+ 20 μM RA, *p <* 0.01; MPTP *vs.* MPTP+ 40 μM RA, *p <* 0.01; MPP^+^ *vs.* MPP^+^+ 80 μM RA, *p <* 0.001; MPP^+^ *vs.* MPP^+^+ 100 μM RA, *p <* 0.001; Figure 4A). To evaluate oxidative stress, we measured the activity of ROS. MPP+ increased the green/red fluorescence intensity in the SH-SY5Y cells (MPP^+^ *vs.* control, *p <* 0.05; Figure 4C) and these changes were significantly reversed by RA in a dose-dependent manner (MPP^+^ *vs.* MPP^+^+ 60 μM RA, *p <* 0.05; MPP^+^ *vs.* MPP^+^+ 80 μM RA, *p <* 0.05; MPP^+^ *vs.* MPP^+^+ 200 μM RA, *p <* 0.05; Figure 4B). Therefore, we further evaluated mitochondrial function after MPP+ and RA administration. JC-1 was then utilized to detect mitochondrial membrane potential (MMP). As expected, the green/red fluorescence intensity increased in the MPP_+_ group when stained with JC-1 (MPP^+^ *vs.* control, *p <* 0.001; MPP^+^ *vs.* PBS, *p <* 0.001; Figure 5B), and this ratio was markedly decreased by preincubation with RA (MPP^+^ *vs.* MPP^+^+ 20 μM RA, *p <* 0.01; MPP^+^ *vs.* MPP^+^+ 40 μM RA, *p <* 0.001; MPP^+^ *vs.* MPP^+^+ 60 μM RA, *p <* 0.001; MPP^+^ *vs.* MPP^+^+ 80 μM RA, *p <* 0.001; MPP^+^ *vs.* MPP^+^+ 100 μM RA, *p <* 0.001; MPP^+^ *vs.* MPP^+^+ 200 μM RA, *p <* 0.001; Figure 4C).

### RA Alleviated MPTP/MPP^+^-Induced Mitochondrial Damage *via* the mtUPR

To explore whether the treatment with RA is related with the mtUPR, Western blotting was used to detect the expression of molecular chaperones and proteases in the mtUPR proteins, including CLPP, LONP1, HSAP9, and HSPE1. 1) An obvious increase was observed in the expression of CLPP in the MPP^+^ group compared with the control and PBS (control *vs.* MPP^+^, *p <* 0.001; PBS *vs.* MPP^+^, *p <* 0.001; Figure 5A), which was significantly improved with the pre-administration of 40, 60, 80, 100 or 200 μM RA (MPP^+^ *vs.* MPP^+^+ 40 μM RA, *p <* 0.01; MPP^+^ *vs.* MPP^+^+ 60 μM RA, *p <* 0.05; MPP^+^ *vs.* MPP^+^+ 80 μM RA, *p <* 0.001; MPP^+^ *vs.* MPP^+^+ 100 μM RA, *p <* 0.001; MPP^+^ *vs.* MPP^+^+ 200 μM RA, *p <* 0.001; Figure 5A). 2) Compared with the control and PBS, MPP^+^ led to an increase in the expression of LONP1 (control *vs.* MPP^+^, *p <* 0.001; PBS *vs.* MPP^+^, *p <* 0.001; Figure 5B), while the pre-injection of 40, 60, 80 or 100 μM RA reduced this increase significantly (MPP^+^ *vs.* MPP^+^+ 40 μM RA, *p <* 0.01; MPP^+^ *vs.* MPP^+^+ 60 μM RA, *p <* 0.001; MPP^+^ *vs.* MPP^+^+ 80 μM RA, *p <* 0.001; MPP^+^ *vs.* MPP^+^+ 100 μM RA, *p <* 0.001; Figure 5B). 3) The expression of HSPA9 in the MPP^+^ group obviously increased compared with the control and PBS (control *vs.* MPP^+^, *p <* 0.001; PBS *vs.* MPP^+^, *p <* 0.001; Figure 5C), while the pre-administration of 40, 60, 80, 100 or 200 μM RA inhibited this increase significantly (MPP^+^ *vs.* MPP^+^+ 40 μM RA, *p <* 0.05; MPP^+^ *vs.* MPP^+^+ 60 μM RA, *p <* 0.001; MPP^+^ *vs.* MPP^+^+ 80 μM RA, *p <* 0.001; MPP^+^ *vs.* MPP^+^+ 100 μM RA, *p <* 0.001; MPP^+^ *vs.* MPP^+^+ 200 μM RA, *p <* 0.001; Figure 5C). 4) An obvious increase was observed in the expression of HSPE1 in the MPP^+^ group compared with the control and PBS (control *vs.* MPP^+^, *p <* 0.001; PBS *vs.* MPP^+^, *p <* 0.01; Figure 5D), which was significantly inhibited with the pre-administration of 20, 40, 60, 80, 100 or 200 μM RA (MPP^+^ *vs.* MPP^+^+ 20 μM RA, *p <* 0.05; MPP^+^ *vs.* MPP^+^+ 40 μM RA, *p <* 0.001; MPP^+^ *vs.* MPP^+^+ 60 μM RA, *p <* 0.01; MPP^+^ *vs.* MPP^+^+ 80 μM RA, *p <* 0.001; MPP^+^ *vs.* MPP^+^+ 100 μM RA, *p <* 0.001; MPP^+^ *vs.* MPP^+^+ 200 μM RA, *p <* 0.01; Figure 5D).

**FIGURE 6:**
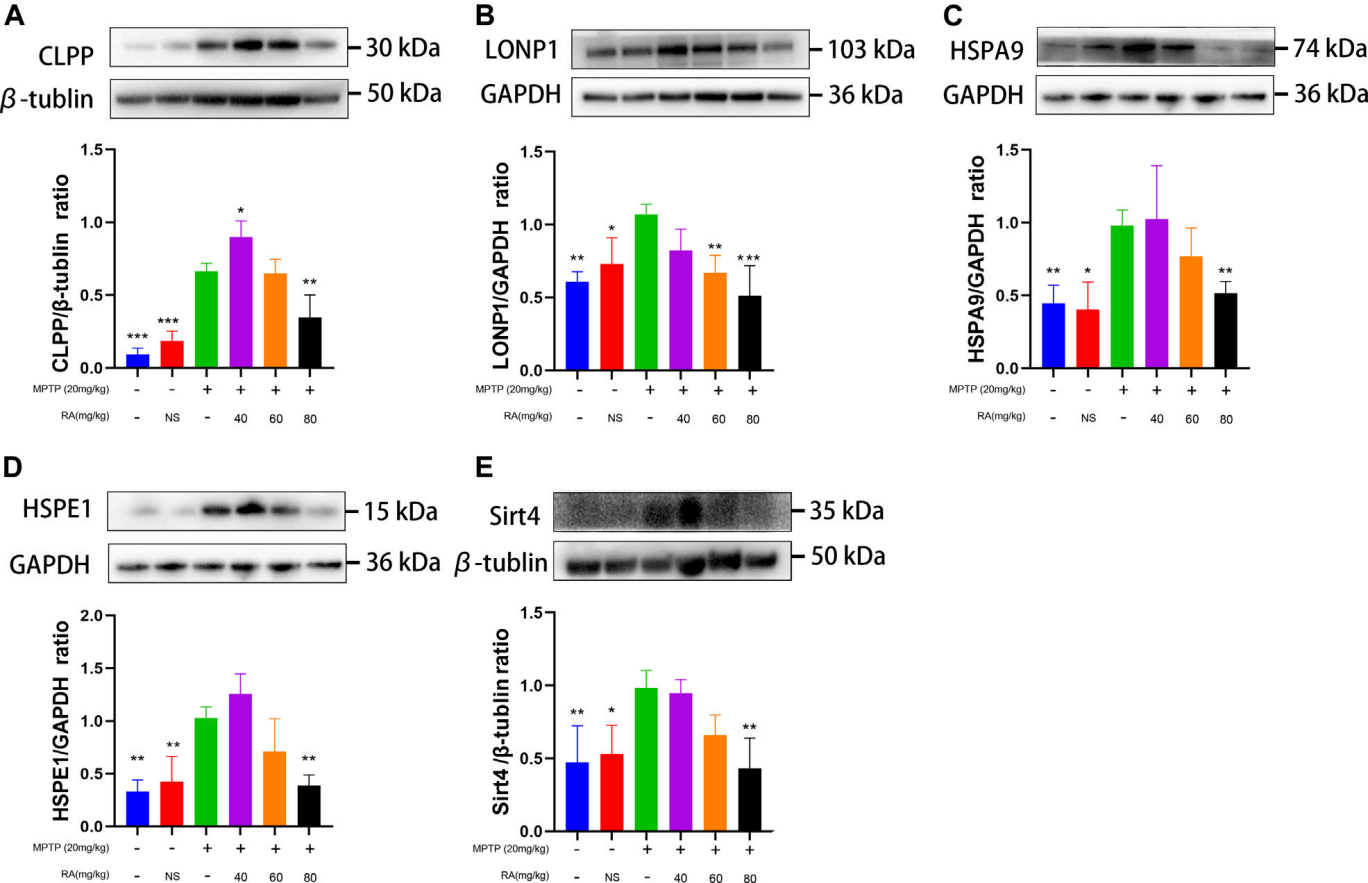
RA alleviated MPTP-induced mitochondrial damage via the mtUPR in MPTP-mice. MPTP caused a significant increase in CLPP **(A)**, LONP1 **(B)**, HSPA9 **(C)**, and HSPE1 **(D)** expression, and RA reversed these increases in a dose-dependent manner. RA regulated SIRT4 **(E)** expression in MPTP-mice. The expression of SIRT4 increased by MPTP administration, and RA reversed this increase in a dose-dependent manner. Data are expressed as mean ± SD. One-way analysis of ANOVA with Tukey's test was used in CLPP, LONP1, HSPE1, and Sirt4. One-way analysis of ANOVA with Dunnett's T3 test was used in HSPA9. N = 4 mice per group, *p < 0.05, **p < 0.01, ***p < 0.001 when compared with the MPTP group.

For further verification, we explored the molecular mechanism of RA alleviating MPTP-induced SN mitochondrial dysfunction. 1) A significant increase was observed in the expression of CLPP in the SN after injecting with MPTP (control *vs.* MPTP, *p <* 0.001; NS *vs.* MPTP, *p <* 0.001; Figure 6A), while pretreatment with 40 or 80 mg/kg RA significantly reduced this increase (MPTP *vs.* MPTP+ 40 mg/kg RA, *p <* 0.05; MPTP *vs.* MPTP+ 80 mg/kg RA, *p <* 0.001; Figure 6A). 2) A significant increase was observed in the expression of LONP1 in the SN after injecting with MPTP (control *vs.* MPTP, *p <* 0.01; NS *vs.* MPTP, *p <* 0.05; Figure 6B), which was significantly inhibited with the pretreatment with 60 or 80 mg/kg RA (MPTP *vs.* MPTP+ 60 mg/kg RA, *p <* 0.01; MPTP *vs.* MPTP+ 80 mg/kg RA, *p <* 0.001; Figure 6B). 3) An increased protein expression of HSAP9 in the SN was observed after injecting with MPTP (control *vs.* MPTP, *p <* 0.01; NS *vs.* MPTP, *p <* 0.05; Figure 6C), and this increase was reversed by the pre-administration of 80 mg/kg RA (MPTP *vs.* MPTP+ 80 mg/kg RA, *p <* 0.01; Figure 6C). 4) Compared with the control and PBS, MPTP led to an increase in the expression of HSPE1 in the SN (control *vs.* MPTP, *p <* 0.01; NS *vs.* MPTP, *p <* 0.01; Figure 6D), while pretreatment with 80 mg/kg RA significantly reduced this increase (MPTP *vs.* MPTP+ 80 mg/kg RA, *p <* 0.01; Figure 6D).

For exploring whether RA alleviated MPTP/MPP^+^-induced mitochondrial damage via sirtuin signaling. The SH-SY5Y cells were administered with MPP_+_ and various concentrations of RA. Increased protein expression of SIRT4 was observed in the MPP^+^ group compared with the control (control *vs.* MPP^+^, *p <* 0.05; Figure 5E), and this increase was reversed by the pre-administration of 80, 100 or 200 μM RA (MPP^+^ *vs.* MPP^+^+ 80 μM RA, *p <* 0.05; MPP^+^ *vs.* MPP^+^+ 100 μM RA, *p <* 0.05; MPP^+^ *vs.* MPP^+^+ 200 μM RA, *p <* 0.05; Figure 5E). For further verification, a significant increase was observed in the expression of SIRT 4 in the SN after injecting with MPTP (control *vs.* MPTP, *p <* 0.01; NS *vs.* MPTP, *p <* 0.05; Figure 6E), while pretreatment with 80 mg/kg RA significantly reduced this increase (MPTP *vs.* MPTP+ 80 mg/kg RA, *p <* 0.01; Figure 6E).

## Discussion

Most researchers believe that the mitochondrial damage of dopaminergic neurons is important to the pathogenesis of PD, the damage and protective mechanism are still controversial (Hu et al., 2019; Trist et al., 2019; Vasquez et al., 2020; Malpartida et al., 2021). mtUPR is the first stress-protective response induced by mitochondrial damage; mtUPR repairs or removes the misfolded proteins to alleviate the damage (Qureshi et al., 2017; Ji et al., 2020). Studies have confirmed that the Sirtuin family of proteins is essential for mitochondrial stress response (Liang and Ghaffari, 2017). In this study, we found that mtUPR is activated in the MPTP-induced damage of dopaminergic neurons in mice. RA may maintain the mitochondrial homeostasis of dopaminergic neurons through mtUPR and Sirtuin, which provides novel insights for developing functional food supplements and preventive or therapeutic drugs for neurodegenerative diseases and other mitochondria-related diseases.

MPTP is an internationally recognized synthetic toxin that can induce Parkinson-like symptoms in humans, primates, and mice. An acute PD model was established by i.p. injection of 20 mg/kg MPTP every 2 h for 4 times (Kuroiwa et al., 2010; Bov é and Perier, 2012). The results show that the overall motor ability (pole test, rotarod test, and the total distance and average speed of open field test) of the MPTP group was significantly lower than that of the control group (Figure 1), so was the TH neurons, indicating successful modeling of PD (Figure 2). There is no good correlation between the degree of TH neuron injury and behavior in this experiment. We speculate that RA has a supporting therapeutic effect, the neuronal protective effect is not dose-related, and the symptom-improving effect is dose-related. For example, Madopa as a clinical drug, it can improve symptoms, but there is no change in TH neurons. Ultrastructural pathological sections show prominent structural abnormalities in the mitochondria of dopaminergic neurons of the MPTP group (Figure 3B). The proportion of class III-type mitochondria in the MPTP group increased significantly (Figure 3C).

Previous studies in our lab proved that one of the mechanisms of MPTP-induced PD-like damage to mice or cells is dopaminergic neurons’ oxidative stress response, closely associated with mitochondrial damage (Wang et al., 2019; Cai et al., 2020). Mitochondrial damage can lead to the imbalance of mitochondrial PQC, resulting in PD (Liang and Ghaffari, 2017). mtUPR is the earliest stress response in the entire mitochondrial PQC system (Weng et al., 2020), in fact, a reverse signal transduction process from mitochondria to nucleus (Fiorese and Haynes, 2017). The production (quantity and degree) of molecular chaperones encoded by the nucleus (e.g., HSPA9, HSPE1, and HSPD1) and proteases (e.g., CLPP, YME1L1, and LONP1) are currently considered as biomarkers of mtUPR activation (Weng et al., 2020). Their function is to timely correct the imbalance of protein homeostasis under stress conditions to prevent further damage, thus maintaining the best quality and function of the mitochondrial protein and ensuring the normal activity of cells (Ferreiro et al., 2012; Jovaisaite and Auwerx, 2015; Smyrnias et al., 2019; Ji et al., 2020).

This study found that dopaminergic neurons’ level of mtUPR markers (HSPA9, HSPE1, CLPP, and LONP1) significantly increased in the MPTP group (Figures 5A–D), and the level of SIRT4 also increased. Dopaminergic neuron mtUPR was activated in the SN at the early stage of MPTP-induced damage, representing a series of dopaminergic neuron damage and further behavioral abnormalities in MPTP-treated mice.

In Figure 4, while the JC-1 ratio and ROS induction assay indicate the RA at 200 μM is protective against mitochondrial stress, it fails to rescue the MPP^+^-induced cell damage in the viability test. Our speculate that CCK8 indicators cannot truthfully represent cell activity in MPP^+^ treated SH-SY5Y cells receiving 200 μM RA. Because RA per se at 200 μM may be toxic to SH-SY5Y. The mechanism may not be related to mitochondria, and further studies are needed.

Many studies have reported the protective effect of polyphenols in neurodegenerative diseases (Devi and Chamoli, 2020; Ebrahimpour et al., 2020; Rebas et al., 2020; Wei et al., 2020). Polyphenols are among the most studied natural compounds ubiquitous in plants and foods, such as herbs, nuts, vegetables, fruits, and plant beverages (including tea and coffee). RA is a phenolic compound usually present in various Labiatae plants (Mentha). We have conducted many studies on polyphenols (Ye et al., 2012a; Ye et al., 2012b; Ye et al., 2016; Wang et al., 2019), including the extraction and toxicity of RA, as well as an in-depth study of the anti-inflammatory activity of RA (Wei et al., 2018; Wei et al., 2021).

In this study, we found that the treatment of MPTP-treated mice with RA (80 mg/kg) improved their behavior (Figure 1), significantly increased the survival rate of dopaminergic neurons (Figure 3F) and restored the mitochondrial structure to normal (Figures 3B–D). The proportion of class I mitochondria recovered to the same level as the control group (Figure 3C). These results indicate that RA can protect dopaminergic neurons from MPTP toxicity, possibly through the protection of mitochondria. At the same time, RA decreased the levels of mtUPR markers (HSPA9, HSPE1, CLPP, and LONP1) in the dopaminergic neurons of MPTP-treated mice (Figures 6A–D). RA is a natural polyphenol with broad-spectrum biological activity (Wei et al., 2021; Wei et al., 2018). Other studies have found that RA can prevent the damage of neuronal mitochondria by increasing the number of mitochondria and protecting the morphological structure of mitochondria (Qu et al., 2019); RA also has a protective effect on dopaminergic neurons (Ren et al., 2009; Zhao et al., 2020), consistent with our findings. Further study showed that the protein expression of SIRT 4 decreased after RA treatment (Figure 6E), indicating that RA may maintain the mitochondrial homeostasis of dopaminergic neurons via Sirt. The SIRT family comprises seven proteins (SIRT 1–7), which can participate in various cellular processes (Vassilopoulos et al., 2011; Weng et al., 2020). In the Sirtuin family, SIRT 1, SIRT6, and SIRT7 are mainly present in the nucleus; SIRT3, SIRT4, and SIRT5 are mainly present in the mitochondria; and SIRT2 is mainly present in the cytoplasm. It is demonstrated that mtUPR may be an important component of SIRT-mediated longevity function (Nasrin et al., 2010). The expression and activity of SIRT4 are related to the susceptibility of neurodegenerative diseases (van de Ven et al., 2017; Lautrup et al., 2019). There are few related studies on PD models.

RA is increasingly used in the cosmetic and food industry; cosmetics and functional foods containing RA are sold (Naimi et al., 2017; Ngo et al., 2018). Moreover, RA is a potential natural drug for protecting neurodegenerative diseases.

In conclusion, our study shows that RA can restore the mitochondrial homeostasis of dopaminergic neurons in MPTP-treated mice through mtUPR, correct the unfolded protein response, prevent the damage of dopaminergic neurons, and preserve mitochondrial morphology, thus reversing the MPTP-induced PD-like motor decline in mice. Therefore, this study helps develop RA as a functional food supplement, provides novel insights, and suggests that RA is a potential natural preventive or therapeutic drug for neurodegenerative diseases.

## Data Availability

The original contributions presented in the study are included in the article/Supplementary Material, further inquiries can be directed to the corresponding authors.
